# Information systems adoption and knowledge performance: An absorptive capacity perspective

**DOI:** 10.3389/fpsyg.2022.1062780

**Published:** 2023-01-10

**Authors:** Huiyan Liao, Yi Liu, Peigong Li

**Affiliations:** ^1^School of Management, Xiamen University, Xiamen, China; ^2^H-E-B School of Business and Administration, University of the Incarnate Word, San Antonio, TX, United States; ^3^School of Accounting, Shanghai Lixin University of Accounting and Finance, Shanghai, China

**Keywords:** information systems adoption, absorptive capacity, knowledge sharing, organizational knowledge performance, 4th IR

## Abstract

As strategic assets for organizations, information systems (IS) have been adopted to enhance organizational knowledge performance. Based on the absorptive capacity perspective, we investigated intertwined relationships among IS adoption, organizational capabilities, IS-enabled absorptive capacity, and organizational knowledge performance. We empirically examined our model with survey data from 417 IS employees of 21 different state governments in the United States. We find that: (1) IS adoption does not directly generate IS-enabled absorptive capacity; (2) organizational capabilities positively affect IS-enabled absorptive capacity; (3) synergies arising from complementarity between IS adoption and organizational capabilities have a positive impact on IS-enabled absorptive capacity; and (4) IS-enabled absorptive capacity significantly drives manager and employee knowledge performance. This research enriches the understanding of the relationships among IS adoption, organizational capabilities, and organizational knowledge performance in U.S. public sectors.

## Introduction

In the big data era, fast-paced environmental changes require that organizations take proactive actions to improve their innovation performance which is considered imperative for organization success (Arena et al., 2017). Organizations in private sectors provide products or services to consumers in the market place to earn profits for shareholders while organizations in public sectors provide products or services to consumers in the needs of people that call for a public response (Nutt, 2006). Public sectors, such as state governments, are facing a myriad of challenges (e.g., budget cuts, service expansions, and political turmoil) in addition to the constant and rapid technological changes faced by private sector firms (McHugh, 1997; Micheli et al., 2012). These challenges shape innovation performance of organizations in public sector. Thus, how to improve innovation performance is a critical issue to decision makers of organization in public sector.

Previous studies proposed that organizational innovation is a commercial end of knowledge (Cohen and Levinthal, 1989). Organizational knowledge can lead an organization towards a sustainable competitive advantage, and is one of the most important factors that influences organizational innovation (Nouri et al., 2017; Haneda and Ito, 2018). Thus, organizational knowledge performance influences innovation performance (Cohen and Levinthal, 1990). To improve organizational knowledge performance, scholars proposed that absorptive capacity is a knowledge-based capability of using knowledge from outside organizational boundaries to facilitate organization knowledge performance (Matusik and Heeley, 2005; Tu et al., 2006). Those scholars found that absorptive capacity is one of the key factors in improving organizational knowledge performance as well as innovation (Cohen and Levinthal, 1989; Cohen and Levinthal, 1990; Riemenschneider et al., 2010; Liu et al., 2018).

Scholars argued that gaining knowledge from external is the key to facilitate the absorptive capacity (Lichtenthaler and Lichtenthaler, 2009; Roberts et al., 2012). Previous studies found that information systems can help organizations gain knowledge from external business environment (Roberts et al., 2016; Liu et al., 2021). To improve organizational knowledge performance and innovation, state governments need to adopt information systems to gather information about advanced technologies and enhance the knowledge of their employees. Those information systems, which refers to computer-based systems designed to collect, process, store, and distribute information, such as enterprise resource systems (ERP), customer relationship management systems (CRM), database management systems (DBMS) (Boaden and Lockett, 1991; Piccoli, 2007), plays a critical role in knowledge performance (Pérez-López and Alegre, 2012; Trantopoulos et al., 2017).

However, the challenge for state government is that information systems that may be perfectly adequate for use in a business with a limited set of strategic goals may not work in a state government environment populated by many independent agencies that must respond to a diverse set of stakeholders (Harvey et al., 2010). In addition, constitutional and legal constraints may affect IS adoption of state government (Liu et al., 2018). However, few studies explore the relationship between IS adoption, absorptive capacity, and organizational knowledge performance in public sectors. Thus, it is important for decision makers to understand the relationships among IS adoption, absorptive capacity, and organizational performance in public sector context. Therefore, we attempt to examine the following two research questions:

*RQ1*: How do IS adoption and organizational capabilities drive IS-enabled absorptive capacity in state government IS de\partments?

*RQ2*: How does IS-enabled absorptive capacity influence state government knowledge performance on employee level?

We draw on the absorptive capacity perspective to understand the intertwined relationship among IS adoption, organizational capabilities, and knowledge performance. By doing so, we are able to extend absorptive capacity in a brand-new context and provide actionable insights to state governments to help them uncover the factors that may lead to increased organizational knowledge performance.

## Literature review

Absorptive capacity refers to a firm’s ability to identify, assimilate, transform, and apply valuable external knowledge (Cohen and Levinthal, 1990; Roberts et al., 2012). It is knowledge-based capacity, which has four components: acquisition, assimilation, transformation, and exploitation (Zahra and George, 2002; Riemenschneider et al., 2010; Liu et al., 2018). The acquisition is the ability to identify and acquire knowledge from external. Assimilation is the ability to analyze, process, interpret, and understand knowledge. Transformation is the ability to develop and refine the routines for combining existing knowledge and newly acquired knowledge. Exploitation is the ability to leverage the existing knowledge and integrate new knowledge such that it may be applied in the firm. Scholars further propose that absorptive capacity can be divided into two types: potential absorptive capacity, and realized absorptive capacity (Zahra and George, 2002; Patel et al., 2015). Potential absorptive capacity makes the firm receptive to acquiring and assimilating external knowledge (Lane and Lubatkin, 1998) while realized absorptive capacity is the firm’s capacity to leverage the knowledge that has been absorbed (Zahra and George, 2002). Since organization innovation is the commercialized knowledge of an organization (Cohen and Levinthal, 1990), absorptive capacity has been identified as an important factor that influences organizational knowledge output and innovation (Dong and Yang, 2015; Engelman et al., 2017; Gao et al., 2017; Ali et al., 2018; Zou et al., 2018).

Because absorptive capacity is considered imperative for organizational knowledge output and innovation, many studies focus on understanding antecedents of absorptive capacity. Previous studies find that absorptive capacity could be influenced by organizational capabilities (Van den Bosch et al., 1999; Jansen et al., 2005; Armstrong et al., 2015), and external knowledge resources and sharing (Kostopoulos et al., 2011; Marabelli and Newell, 2014; Flor et al., 2018). Since external knowledge and market information are important in absorptive capacity (Hodgkinson et al., 2012; Flor et al., 2018), researchers claim that inter-organizational networks play an important role in determining absorptive capacity (Agramunt et al., 2020). An organization can produce more innovations and enjoy better performance if it can absorb new knowledge from other units (Tsai, 2001). These findings support that knowledge source determines absorptive capacity, which in turn determines organizational knowledge outcome (Todorova and Durisin, 2007).

Given that modern information technologies perform a critical role in the organization, scholars point out that IS resources and organizational resources together impact absorptive capacity (Francalanci and Morabito, 2008; Bolívar-Ramos et al., 2013; Setia and Patel, 2013). Roberts et al. (2012) argue that the rapid convergence and diffusion of computing, communications, and content technologies offer firms significant opportunities in enhancing absorptive capacity. For instance, information systems help inter-organizational information exchanges (Malhotra et al., 2005; Iyengar et al., 2015), and provide high accessibility to accurate, comprehensive, and timely market information (Jimenez-Castillo and Sanchez-Perez, 2013). This enables the organization to exchange and process knowledge with low technologic constraints (Liu et al., 2013), offer electronic repositories for obtaining and accumulating relationship-specific knowledge developed through inter-organizational relationships (Choi, 2014), and support business processes redesign (Manfreda et al., 2014). By doing so, information systems positively influence organizational absorptive capacity. The relationship between IS and absorptive capacity has been further supported by IS studies focusing on one or more specific IS characteristics in organizations (Chang et al., 2013; Teigland et al., 2014; Liu et al., 2018).

Notwithstanding the importance of absorptive capacity and its related antecedents in literature, there is a call to develop this theory. Roberts et al. (2012) indicate that organizational capabilities and organizational IS resources are two key drivers of absorptive capacity and argue that a possible theoretical contribution from further research should be built on the intertwined relationship between IS and organizational capabilities. To respond to these calls, we focus on organizational capabilities and IS adoption. In this study, we define IS adoption as to what extent an organization utilizes its technology resources. IS-enabled absorptive capacity refers to information systems supporting firms’ ability of knowledge acquisition, assimilation, transformation, and exploitation (Liu et al., 2018). Organizational capabilities refer to “a high-level routine (or set of routines) that confers a set of decision options on an organization’s management for producing significant outputs of a particular type” (Winter, 2003). Organizational capabilities have two sub-dimensions: social capabilities and coordination capabilities (Roberts et al., 2012). Social capabilities refer to a firm’s ability to produce a shared ideology that offers organizational members an attractive identity as well as collective interpretations of reality (Van den Bosch et al., 1999) while coordination capabilities refer to a firm’s ability to manage the dependencies among its various activities (Roberts et al., 2012). In addition, knowledge performance is defined as the degree to which an individual is knowledgeable about a specific domain (Mannucci and Yong, 2018). In an organization, knowledge performance increases the complexity of knowledge structures and determines organizational creativity and innovation performance (Mannucci and Yong, 2018). In this paper, we regard knowledge performance into two types: managerial knowledge performance and non-managerial knowledge performance. We define manager knowledge performance as to what extent the managerial team understand job skills, technology and practices possessed in the organization while employee knowledge performance is defined as to what extent the non-managerial employees understand job skills, technology and practices possessed in the organization (Armstrong et al., 2015).

## Hypotheses development

### IS adoption and IS-enabled absorptive capacity

Information systems (IS) have been identified as the key resources to explore knowledge from external. By adopting information technologies, Organizations can build the relationship with external and collect knowledge from the external environment. For example, online feedback systems or customer relationship management systems enable organizations to identify and exchange valuable knowledge from customers, thus facilitating organizational knowledge identification capability. In addition, by adopting IS, organizations can increase knowledge application capability. For example, integration systems (e.g., ERP, SOA) provide immediate access to standardized data across organizational units, which in turn allow the organization to more readily apply new knowledge to create and provide products and services (Roberts et al., 2012). Moreover, by adopting IS, organizations can facilitate their knowledge assimilation/transformation capability. For example, knowledge management systems enable storing, archiving, retrieving, and sharing of current knowledge to gain a better understanding of how new external knowledge relates to what organizational members already know (Roberts et al., 2012). Similarly, service-oriented architecture (SOA) helps organizations interpret knowledge received from others, thereby enhancing knowledge assimilation. Thus, we propose the following hypothesis:

*H1*: IS adoption is positively related to IS-enabled absorptive capacity.

### Organizational capabilities and IS-enabled absorptive capacity

Organizational capabilities have two dimensions: socialization capabilities and coordination capabilities (Roberts et al., 2012). Socialization capabilities include shared languages, and shared goals (Roberts et al., 2012). With higher socialization capabilities, organizations enable to create strong, understandable, manager-supported, and widely shared values, ensuring that individuals are connected to broader organizational goals. This can strengthen alignment between individual values and the organization’s ideology (Paarlberg and Lavigna, 2010), and thus lead to knowledge transfer and exchanges (Inkpen and Tsang, 2005). Coordination capabilities are the ability to manage the dependencies among organizational various activities (Roberts et al., 2012) and they not only enhance knowledge exchanges between organization and external environment but also increase knowledge exchanges among individuals within organizations (Kotha et al., 2013). Taken together, with higher organizational capabilities, employees in the organization are more likely to identify, assimilate, transfer and apply knowledge. Therefore, we propose the following hypothesis:

*H2*: Organizational capabilities are positively related to IS enabled absorptive capacity.

### The synergies between is adoption and organizational capabilities

IS adoption and organizational capabilities jointly act in concert to enhance IS-enabled absorptive capacity. Their mutually reinforcing interplay allows employees in organization to better knowledge acquisition, assimilation, transformation, and exploitation. For example, information systems may help firms to collect information from external. However, simply adopting information systems in organization may not significantly impact the employees’ ability to identify valuable knowledge. Organizational capabilities can identify the flow of valuable knowledge into the organization (Roberts et al., 2012). In addition, information gained by adopted information systems is usually in “raw” form, which is not ready for immediate use by the organization (Roberts et al., 2012). Organizational capabilities help the firm assimilate and transform raw data into useful knowledge (Jansen et al., 2005). In addition, organizational capabilities can facilitate knowledge sharing and exchange. Information systems (e.g., SOA, knowledge management systems) can accelerate knowledge sharing and exchange process. Moreover, transferring knowledge among employees or departments may have barriers due to the various backgrounds or business functionalities. Organizational capabilities can create a shared goal and language among different parties, which speeds up knowledge transfer and exchanges within the organization. Thus, by combining IS adoption with organizational capabilities, organization can enhance knowledge assimilation and transformation. Therefore, we propose the following hypothesis:

*H3*: Synergies arising from complementarity between IS adoption and organizational capabilities are positively related to IS-enabled absorptive capacity.

### Is-enabled absorptive capacity and organizational knowledge performance

Although absorptive capacity has been primarily used to explain firm level phenomena, a firm’s absorptive capacity depends on the absorptive capacities of its individual members (Liu et al., 2018). It is comprised of the knowledge of employees and managers in the organizations. Individuals with higher levels of absorptive capacity can effectively explore external knowledge (Lichtenthaler and Lichtenthaler, 2009), gain a better understanding of the new knowledge from external (Chen, 2004; Lichtenthaler and Ernst, 2012), transfer substantial inbound knowledge transfer (Lichtenthaler and Lichtenthaler, 2010), and thus incorporate it into their knowledge base (Zahra and George, 2002; Mahnke et al., 2005). The expanded knowledge base determines the volume of knowledge that may be shared and exploited by individuals. Sharing and exploiting knowledge enables individuals to see things from different perspectives, discover new questions to be answered, and thus expand their knowledge base (Zhu et al., 2018), and help individuals to enhance their knowledge performance (Lublin, 2003; Zhu et al., 2018; Pian et al., 2019). Thus, we propose the following hypotheses:

*H4a*: IS-enabled absorptive capacity is positively related to managers’ knowledge performance.

*H4b*: IS-enabled absorptive capacity is positively related to employees’ knowledge performance.

## Methodology

### Research design

**Figure 1:**
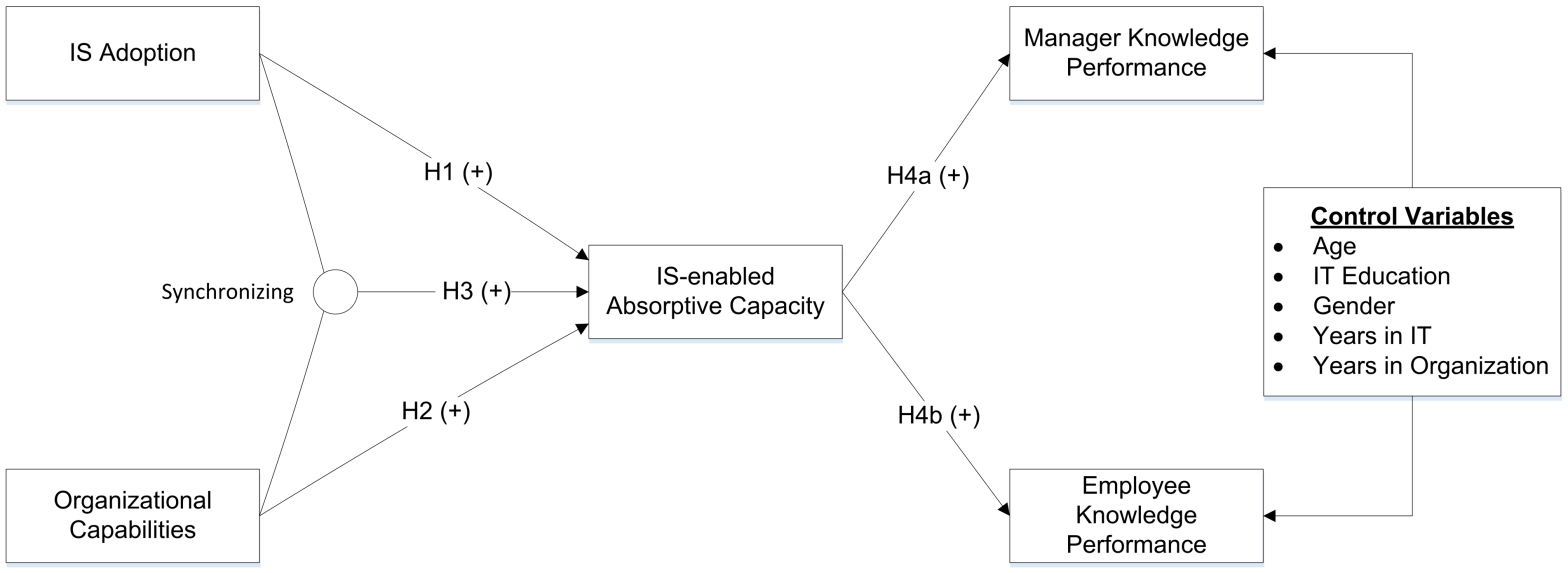
Research Model.

In this study, we propose that IS adoption and organizational capabilities are two factors that independently and jointly affect IS-enabled absorptive capacity, which increases both manager and employee knowledge performance. Since individual cognitions are the basis of absorptive capacity (Lane et al., 2006), five control variables were included in the model to exclude potential noise caused by individual differences. Those five control variables are employees’ age, IS education, gender, years in IS field, and years in their organization. Previous studies found that an employee’s age and gender shape his/her cognition (He et al., 2007; Angst and Agarwal, 2009), and therefore we control age and gender. Work experience and the level of academic degree are two important factors that also influence absorptive capacity (Lund Vinding, 2006). Thus, we include those five variables as control variables. Figure 1 presents our research model.

### Measurement development

We collect survey data from IT departments within U.S. state governments for two reasons. First, state government agencies may face an environment that has stringent rules and procedures that can make it hard to react swiftly in an era of rapid technological changes. Second, compared to the private sector, the low attractiveness of incentive scheme of state government agencies often makes it hard to recruit and retain talents. Thus, it is critical for state government agencies to know how to improve their knowledge performance. The survey items were adapted or adopted from previously validated scales and were measured using a 7-point Likert scale unless specified otherwise. All of the constructs were operationalized as reflective indicators. In this study, we explored the adoption of three common IS assets: customer relationship management systems (CRM), service-oriented architecture (SOA), and web 2.0. We capture IS adoption by asking employees to self-evaluate the adopting of an IS asset in their organization (Somers and Nelson, 2004). We measure the perceptions that an individual may have regarding adopting an IS asset for two reasons. First, individuals’ behavior toward the asset is a function of how they perceive the asset. As classic IS theories such as the technology acceptance model consistently demonstrated, perceptions about technology are instrumentals in the adoption decision and ultimately usage behavior (Fishbein and Ajzen, 1975; Davis, 1989). Second, research has found that perceived measures of concepts frequently correlate positively with corresponding objective measures (Morris and Dillon, 1997). We assert that investigating the interaction among perceived attributes helps to establish theory (Moore and Benbasat, 1991), and measuring the perceived level of IS asset adoption is appropriate for this context. As mentioned earlier, organizational capabilities can take two forms: socialization capabilities and coordination capabilities. We thus develop measurements of socialization capabilities (Van der Post et al., 1997; Pandey and Rainey, 2006) and coordination capabilities (Pavlou and El Sawy, 2006) from literature. We measure organizational capabilities as second-order constructs with two first-order constructs of socialization capabilities and coordination capabilities. Referring to (Kwok and Gao, 2005; Liu et al., 2018), IS-enabled absorptive capacity is captured by three items to measure whether IT department is able to recognize, assimilate, and apply the value of knowledge received regarding information systems. Employee knowledge performance is measured by four items to capture the general knowledge of the first-line IS employees while manager knowledge performance is measured by four items that capture the knowledge performance of managers in the state government IT departments (Tu et al., 2006; Armstrong et al., 2015).

### Sample characteristics

We collect data using online questionnaire. We collect the names and e-mail addresses of the state CIOs from National Association of State Chief Information Officers (NASCIO) headquarters, which is a premier organization that provides support to state CIOs through information exchange of IS best practices and innovations. The Executive Director of NASCIO contacted the state CIOs by e-mail, giving them the URL for the survey website and encouraging them to distribute the URL for the survey website to his/her IS employees. The sample for this study consisted of 417 non-managerial employees within state government IT departments representing 21 different states.

Non-response error occurs when survey respondents are systematically different from non-respondents (Sivo et al., 2006). To ensure non-response bias was not a concern with this study, we analyze the responder versus non-responder states and find no significant differences in terms of the regions within the U.S. or the state’s ‘grade’ on the Government Performance Project’s Grading the States 2008 Report (Barrett and Greene, 2008) in which grades of A, B+, B, B-, etc. are given to each state, indicating the impact of the non-respondents error is not a concern in this study. A common control variable in the literature is industry. As all survey participants in this study are state government IS employees, it is a natural control for the industry. The demographics for participants are shown in Table 1.

**Table 1:** Demographics for survey participants.

**Concept**	**Values**	**Statistics**	**Concept**	**Values**	**Statistics**
Gender	Male	152	Position Classification	Administrative	15
	Female	214		Professional	105
	Did Not Report	51		Technical	237
Marital Status	Single	109		Did Not Report	60
	Married	257	Annual Salary	Below $25,000	4
	Did Not Report	51		$25,000–$39,999	62
Job Function	Application Programmer	89		$40,000–$54,999	159
	Project Lead	35		$55,000–$69,999	83
	Software Engineer	8		$70,000–$84,999	36
	Systems Analyst	45		$85,000–$99,999	15
	Systems Programmer	24		$100,000 or above	6
	Other	165		Did Not Report	52
	Did Not Report	51	Age		*M* = 46.33, SD = 9.52
Formal degree in IS major (Education)	Yes	184	Years in organization		*M* = 11.17, SD = 8.78
	No	182	Years of IS experience		*M* = 16.65, SD = 9.92
	Did Not Report	51	Years in current job		*M* = 8.29, SD = 6.94

## Results

Partial least squares (PLS) is considered to be an appropriate method when the research objective is prediction and theory development and the model is complex (Hair et al., 2016). Thus, PLS is used for measurement validation and model testing.

### Measurement validation

An exploratory factor analysis (EFA) is conducted, and factors are extracted through principal component analysis (PCA). Table 2 reports the results of the exploratory factor analysis, and Table 3 presents the sample’s descriptive statistics, correlation, and square root of average variance extracted (AVE). We examine the internal consistency and the convergent and discriminant validity of the construct (MacKenzie et al., 2011; Henseler et al., 2015). Cronbach’s α for each of the latent variables exceeds 0.70, suggesting sound reliability. All the retained items have loadings above the recommended cutoff of 0.70 (Table 2) and the average variance extracted (AVE) for each construct exceeds the recommended level of 0.50 (Table 3), suggesting good convergent validity. Also, all the items have higher loadings on their respective constructs than on other constructs (Table 2) and the square root of AVE for each construct is greater than the correlation between each pair of constructs in the model (Table 3). In addition, HTMT Ratio values range from 0.009 to 0.721, which are less than 0.850. These results indicate sound discriminant validity. To check multicollinearity, variance inflation factor (VIF) values are computed for all of the constructs. The range of VIF is from 1.053 to 1.905, which is well below the acceptable threshold of 10, indicating that multicollinearity is less likely to be an issue (Cohen et al., 2003). As a second-order construct, organizational capabilities are made up of two interrelated first-order constructs: coordination capabilities and socialization capabilities. Following existing literature, we modeled organizational capabilities and socialization capabilities as 2 s-order reflective constructs (Roberts et al., 2012). We follow the process prescribed in existing literature to evaluate the measurement model (Hair et al., 2016; Sarstedt et al., 2019). Our empirical results show that both first-order constructs load significantly (*p* < 0.001): the loadings are 0.905 for coordination capabilities and 0.861 for socialization capabilities.

**Table 2:** Cronbach’s α, composite reliability, and item loadings and cross-loadings.

Construct	Items	1	2	3	4	5	6	7
1. Coordination Capabilities (Cronbach’s α = 0.963; CR = 0.968)	COORD3	**0.863**	0.209	0.063	0.145	0.082	0.101	0.051
	COORD4	**0.855**	0.193	0.108	0.115	0.033	0.123	0.065
	COORD2	**0.847**	0.194	0.114	0.160	0.051	0.127	0.046
	COORD6	**0.839**	0.229	0.192	0.064	0.027	0.079	0.083
	COORD8	**0.836**	0.221	0.123	0.190	0.025	0.036	0.092
	COORD5	**0.834**	0.229	0.155	0.106	0.014	0.080	0.051
	COORD7	**0.807**	0.238	0.132	0.138	0.052	0.029	0.071
	COORD1	**0.798**	0.222	0.140	0.162	0.027	0.125	0.077
2. Socialization Capabilities (Cronbach’s α = 0.927; CR = 0.941)	SC5	0.232	**0.773**	0.238	0.230	0.050	0.092	0.074
	SC3	0.222	**0.761**	0.170	0.292	0.060	0.153	0.041
	SC9r	0.190	**0.760**	0.074	0.129	0.097	−0.030	0.100
	SC1	0.215	**0.759**	0.191	0.247	0.038	0.090	0.026
	SC2	0.160	**0.757**	0.101	0.256	0.024	0.170	0.028
	SC8r	0.230	**0.732**	0.076	0.051	0.007	−0.007	0.047
	SC6r	0.268	**0.692**	0.185	0.040	0.094	−0.072	0.022
	SC4	0.280	**0.655**	0.275	0.240	−0.003	0.063	0.079
3. Employee Knowledge Performance (Cronbach’s α = 0.937; CR = 0.955)	WK1	0.182	0.226	**0.880**	0.186	0.019	0.056	0.030
	WK2	0.171	0.248	**0.867**	0.199	0.039	0.058	0.042
	WK4	0.178	0.253	**0.858**	0.191	0.038	0.101	0.053
	WK3	0.220	0.178	**0.764**	0.180	0.040	0.012	0.087
4. Manager Knowledge Performance (Cronbach’s α = 0.939; CR = 0.957)	MK3	0.230	0.322	0.207	**0.778**	0.075	0.079	0.038
	MK1	0.284	0.364	0.262	**0.761**	0.042	0.077	0.038
	MK4	0.278	0.317	0.265	**0.758**	0.022	0.060	0.033
	MK2	0.246	0.384	0.274	**0.752**	0.017	0.067	0.017
5. Web 2.0-enabled Absorptive Capacity (Cronbach’s α = 0.983; CR = 0.989)	AS3WEB2	0.053	0.086	0.032	0.025	**0.935**	0.166	0.247
	AS2WEB2	0.069	0.076	0.039	0.049	**0.931**	0.173	0.250
	AS1WEB2	0.057	0.074	0.045	0.040	**0.919**	0.188	0.243
6. SOA-enabled Absorptive Capacity (Cronbach’s α = 0.954; CR = 0.970)	AS2SOA	0.165	0.088	0.067	0.061	0.174	**0.912**	0.199
	AS3SOA	0.132	0.104	0.067	0.075	0.167	**0.892**	0.184
	AS1SOA	0.174	0.034	0.059	0.067	0.182	**0.891**	0.155
7. CRM-enabled Absorptive Capacity (Cronbach’s α = 0.964; CR = 0.977)	AS2CRM	0.115	0.076	0.076	0.033	0.255	0.183	**0.911**
	AS3CRM	0.125	0.124	0.083	0.021	0.246	0.201	**0.895**
	AS1CRM	0.124	0.066	0.037	0.039	0.271	0.178	**0.884**

Bold values are represent above 0.600

**Table 3:** Descriptive statistics, correlations, and square root of AVE, (Sample Size: 417).

	1	2	3	4	5	6	7	8	9	10	11	12	13	14	15
1. Age	--														
2. Education	−0.365**	--													
3. Gender	−0.033	−0.082	--												
4. Years in IS	0.588**	−0.079	−0.090	--											
5. YO	0.376**	−0.211**	0.107^*^	0.286**	--										
6. CC	−0.065	0.083	0.092	−0.092	−0.081	**0.891**									
7. SC	−0.007	−0.009	−0.004	−0.086	−0.026	0.557**	**0.815**								
8. CRM	−0.043	−0.105^*^	0.036	−0.109^*^	0.007	0.106^*^	0.250**	--							
9. SOA	0.020	−0.042	0.017	0.052	0.024	0.168**	0.183**	0.575**	--						
10. WEB2	0.021	−0.141**	0.060	−0.023	0.069	0.171**	0.292**	0.627**	0.525**	--					
11. ABCRM	−0.056	0.098	0.202*	−0.153	−0.058	0.462**	0.435**	0.293**	0.052	0.129	**0.966**				
12. ABSOA	−0.080	0.053	0.116	−0.114	−0.060	0.548**	0.425**	0.225^*^	0.382**	0.268**	0.705**	**0.957**			
13. ABWEB2	−0.071	−0.077	0.085	−0.165	0.003	0.298**	0.343**	0.156	0.184^*^	0.222**	0.710**	0.650**	**0.984**		
14. MK	−0.025	−0.001	0.075	−0.083	0.003		0.671**	0.193**	0.155**	0.215**	0.328**	0.452**	0.277**	**0.920**	
15. WK	0.008	−0.072	0.030	−0.079	0.002	0.429**	0.520**	0.179**	0.128*	0.200**	0.348**	0.380**	0.245**	0.581**	**0.918**
Mean	46.33	0.50	0.42	16.65	11.17	4.48	3.65	2.40	2.39	2.59	4.24	4.20	4.07	4.40	4.84
SD	9.52	0.50	0.49	9.92	8.78	1.39	1.34	1.76	1.60	1.80	1.12	1.18	1.10	1.52	1.38
Min	24.00	0.00	0.00	0.00	0.00	1.00	1.00	1.00	1.00	1.00	1.00	1.00	1.00	1.00	1.00
Max	70.00	1.00	1.00	48.00	38.00	7.00	7.00	7.00	7.00	7.00	7.00	7.00	7.00	7.00	7.00

**Correlation is significant at the 0.01 level (2-tailed).

*Correlation is significant at the 0.05 level (2-tailed). The diagonals are the square root of the average variance extracted (AVE) for multi-item constructs. YO, Year in Organization; CC, Coordination Capabilities; SC, Socialization Capabilities; CRM, CRM Adoption; SOA: SOA Adoption; WEB2, Web 2.0 Adoption; ABCRM, CRM-enabled Absorptive Capacity; ABSOA, SOA-enabled Absorptive Capacity; ABWEB2, Web 2.0-enabled Absorptive Capacity; MK, Manager Knowledge Performance; WK, Employee Knowledge Performance.

### Common method bias and endogeneity

Common method bias is assessed after data collection using two tests. First, Harman’s single factor test is used to assess common method bias (Harman, 1976; Podsakoff et al., 2003). Eight factors emerge from the dataset, accounting for 81.95% of the variance and the first factor explains 40.21% of the variance. Then, a partial correlation test is performed using employee turnover as a marker variable to evaluate the impact of common method bias on observed relationships between constructs (Lindell and Whitney, 2001). We correlate a marker variable with the principal constructs and use the smallest positive value to calculate the partial correlation. The results indicate that changes in the partial correlation are statistically nonsignificant. These two tests suggest that common method bias is not an issue for the research.

To alleviate endogeneity concerns caused by omitted variables bias, we have included five control variables. To mitigate the endogeneity issue caused by reverse causality, we build our research model on a solid theory base and let the theory drive our theorizing of the relationships (Rutz and Watson, 2019). Specifically, absorptive capacity literature suggests that synergies arising from complementarity between IS adoption and organizational capabilities create absorptive capacity, which influences organizational performance (Roberts et al., 2012). To alleviate endogeneity concerns caused by measurement errors, we utilize a structural approach that explicitly accounts for the data generating model (and error) based on the theoretical assumptions (Rutz and Watson, 2019).

### Structural model

**Figure 2:**
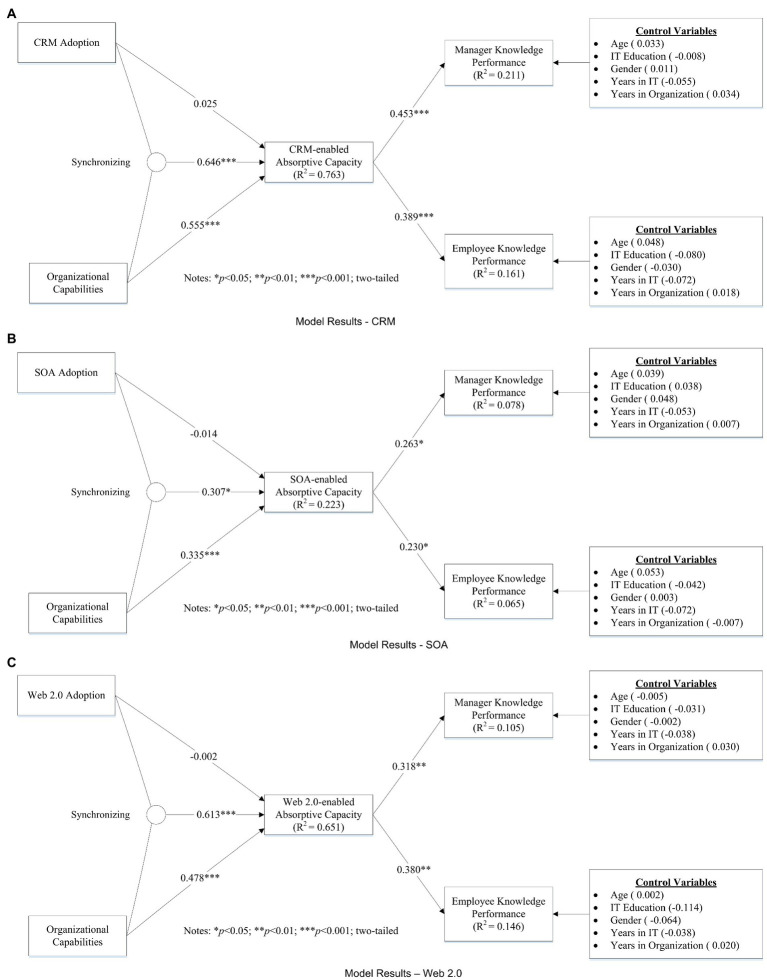
**(A**) Model Results – CRM. **(B)** Model Results – SOA. **(C)** Model Results – Web 2.0.

A standard bootstrap resampling procedure (5,000 samples) is used to evaluate the significance of the paths. The significance of path coefficients is tested using a two-tailed *t*-test. Figure 2 provides the results of the structural model. We test three information systems that are widely adopted in organizations. Results are shown as follows.

**Figure 3:**
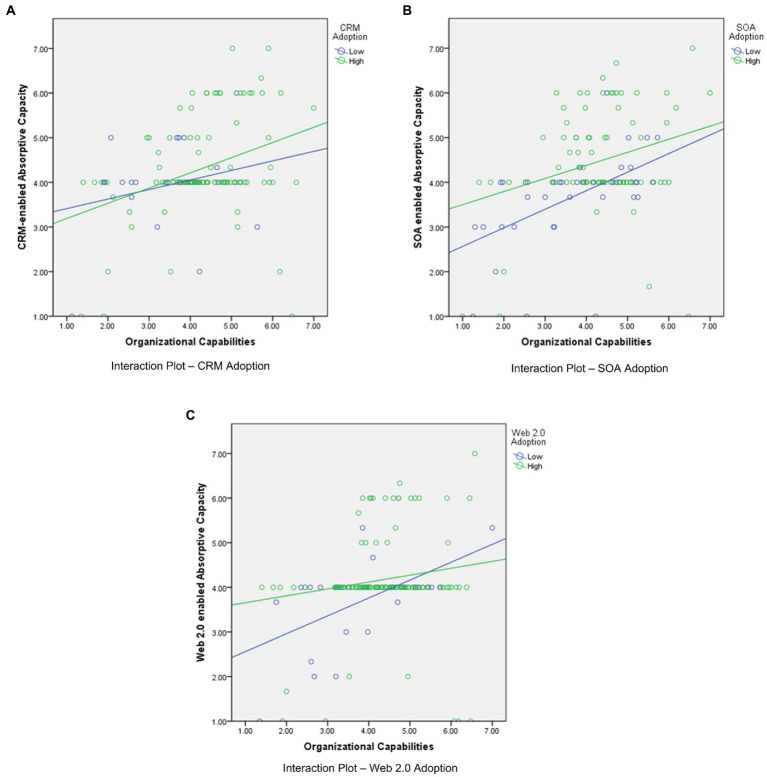
**(A)** Interaction Plot – CRM Adoption. **(B)** Interaction Plot – SOA Adoption. **(C)** Interaction Plot – Web 2.0 Adoption.

For CRM adoption, the model explains 76.3% of the variance in CRM-enabled absorptive capacity, and 21.1% of the variance in manager knowledge performance, and 16.1% of the variance in employee knowledge performance. As Figure 2A shows, we find that (1) CRM adoption is not related to CRM-enabled absorptive capacity directly (H1: *β* = 0.025, *t* = 0.297, *p* > 0.05); (2) organizational capabilities are positively related to CRM-enabled absorptive capacity (H2: *β* = 0.555, *t* = 5.860, *p* < 0.001); (3) synergies arising from complementarity between CRM adoption and organizational capabilities are positively related to CRM-enabled absorptive capacity (H3: *β* = 0.646, *t* = 4.695, *p* < 0.001). Figure 3A visually provides the interaction effect plot; (4) CRM-enabled absorptive capacity is positively related to manager knowledge performance (H4a: *β* = 0.453, *t* = 4.068, *p* < 0.001); and (5) CRM-enabled absorptive capacity is positively related to employee knowledge performance (H4b: *β* = 0.389, *t* = 3.980, *p* < 0.001).

For SOA adoption, the model explains 22.3% of the variance in SOA-enabled absorptive capacity, 7.8% of the variance in manager knowledge performance, and 6.5% of the variance in employee knowledge performance. As Figure 2B shows, we find that (1) SOA adoption is not related to SOA-enabled absorptive capacity directly (H1: *β* = −0.014, *t* = 0.193, *p* > 0.05); (2) organizational capabilities is positively related to SOA-enabled absorptive capacity (H2: *β* = 0.335, *t* = 3.387, *p* < 0.001); (3) synergies arising from complementarity between SOA adoption and organizational capabilities are positively related to SOA-enabled absorptive capacity (H3: *β* = 0.307, *t* = 2.294, *p* < 0.05). Figure 3B visually provides the interaction effect plot; (4) SOA-enabled absorptive capacity is positively related to manager knowledge performance (H4a: *β* = 0.263, *t* = 2.573, *p* < 0.05) and (5) SOA-enabled absorptive capacity is positively related to employee knowledge performance (H4b: *β* = 0.230, *t* = 1.969, *p* < 0.05).

For Web 2.0 adoption, the model explains 65.1% of the variance in Web 2.0-enabled absorptive capacity, 10.5% of the variance in manager knowledge performance, and 14.6% of the variance in employee knowledge performance. As Figure 2C shows, we find support for hypotheses that (1) Web 2.0 adoption is not related to Web 2.0-enabled absorptive capacity directly (H1: *β* = −0.002, *t* = 0.025, *p* > 0.05); (2) organizational capabilities are positively related to Web 2.0-enabled absorptive capacity (H2: *β* = 0.478, *t* = 5.057, *p* < 0.010); (3) synergies arising from complementarity between Web 2.0 adoption and organizational capabilities are positively related to Web 2.0-enabled absorptive capacity (H3: *β* = 0.613, *t* = 4.650, *p* < 0.001). Figure 3C visually provides the interaction effect plot; (4) Web 2.0-enabled absorptive capacity is positively related to manager knowledge performance (H4a: *β* = 0.318, *t* = 2.638, *p* < 0.01); and (5) Web 2.0-enabled absorptive capacity is positively related to employee knowledge performance (H4b: *β* = 0.380, *t* = 2.716, *p* < 0.01).

Above all, these tests indicate that our model supports all hypotheses except H1.

### Robustness check

Three tests are conducted to check the robustness of our results. First, to evaluate the stability of the significance of path coefficients, we use a different number of samples in a bootstrap resampling procedure (6,000 samples) and the new results remain unchanged. Second, we employ a hierarchical regression model to examine whether the interaction of IS (CRM, SOA, and Web 2.0) adoption and organizational capabilities has a substantial impact on IS (CRM, SOA, and Web 2.0) enabled absorptive capacity. The ΔR^2^ resulting from the interaction effect confirms the significance of the interaction effect (Carte and Russell, 2003). Third, even though we do not propose mediation effects in this study, such effects are embedded in our research model. Thus, to examine whether IS-enabled absorptive capacity serves as a mediator between independent variables (IS adoption and organizational capabilities) and dependent variables (manager/employee knowledge performance), we conducted a Sobel test (Sobel, 1982). The statistics of the Sobel test are significant (*p* < 0.05), indicating that IS-enabled absorptive capacity is a mediator between independent variables and dependent variables.

## Discussion

Building on the absorptive capacity perspective, this study develops a research model that aims to reveal the influence of IS adoption and organizational capabilities on organizational knowledge performance in U.S. state government IT departments. By examining IS adoption, this study also answers the call “do the synergies between IT capabilities and complementary capabilities turn into rigidities that eventually create a rigid or narrow absorptive capacity?” (Robert et al., 2012). We confirm that IS adoption does not influence IS-enabled absorptive capacity directly. Instead, Synergies arising from complementarity between IS adoption and organizational capabilities drives IS-enabled absorptive capacity, which facilitates both manager and employee knowledge performance.

### Theoretical implications

This study advances the literature in three ways. **First**, our study contributes to IS adoption literature by examining how synergies arising from complementarity between organizational capabilities and IS assets to impact IS-enabled absorptive capacity. Specifically, previous studies indicated that IS resources play an important role in knowledge exploration (Roberts et al., 2016), which may influence absorptive capacity (Roberts et al., 2012). However, we find that IS assets (CRM, SOA, Web 2.0) cannot influence absorptive capacity directly. Instead, by combining organizational capabilities, IS assets can form IS-enabled absorptive capacity, which can help organization identify, assimilate, transform, and apply valuable external knowledge. In addition, our findings extend previous research by confirming the role of synergies arising from complementarity between organizational capabilities and IS assets may not be consistent. One possible explanation for such variance focuses on different types of IS assets have different functionalities in supporting business process. Thus, we offer a more IS resource-specific understanding of the role of IS adoption in influencing IS-enabled absorptive capacity.

**Second**, this study adds new insights to the absorptive capacity literature by identifying three different IS-enabled absorptive capacities and revealing their roles in organizational knowledge performance. Previous studies in absorptive capacity find absorptive capacity is a factor that influence knowledge sensing, transfer, and innovation (Frank et al., 2015; Roberts et al., 2016). We find that IS-enabled absorptive capacity (CRM-enabled absorptive capacity, SOA-enabled absorptive capacity, and Web 2.0-enabled absorptive capacity) has positive impact on both manager and employee knowledge performance. These findings reveal that IS-enabled absorptive capacity is a key factor that drives organizational knowledge performance of public sectors.

**Third**, organizations worldwide are witnessing and experiencing the 4th Industrial Revolution (4IR), such as artificial intelligence (AI), robotics, the Internet of Things (IoT), etc. The 4IR is considered to be the core driving force for organizations’ innovations and is built on the basis of information systems. Existing studies mainly focus on examining the impacts of those factors in organization in private sectors. However, our paper sheds light on the relation among 4IR, the adoption of AI, RPA, and IoT, organizational capabilities, absorptive capacity, and organizational knowledge performance in state governments. By doing so, we offer a nuanced context-specific understanding of the impacts of those 4^th^ Industrial revolution information systems in organizations in public sectors.

### Practical implications

Corresponding to the theoretical implications mentioned above, this study has three important implications for organizations. First, state governments should develop IS adoption strategies that the acquisition and sharing of knowledge among employees is encouraged and supported. State governments also should understand different roles of IS adoption in supporting their business processes. Second, decision makers should consider the different impact of IS-enabled absorptive capacity on manager and employee knowledge performance. By doing so, decision-makers can leverage appropriate IS assets and combine them with organizational capabilities to generate IS-enabled absorptive capacity, which in turn maximizes manager and employee knowledge performance. Third, in the era of 4IR, when adopting AI, RPA, IoT, 3D printing, or other advanced technologies, government decision-makers can benefit from findings from our paper to enhance absorptive capabilities and gain organizational superior knowledge performance.

### Limitations and future research

As all empirical research, this paper has some limitations, which can be treated as opportunities for further research. **First**, while we tested three major IS assets in the U.S public sections, there are many other IS assets. Considering the 4IR, there are more testable options for advanced technologies. Different advanced tools have different roles in organizations, which may have different impacts on absorptive capacity. Thus, future research could investigate AI, RPA, and IoT in government IT departments and other organizational capabilities to provide more comprehensive understanding. **Second**, in this study, we only examined the impact of IS-enabled absorptive capacity on knowledge performance in U.S public sections. However, knowledge performance has two dimensions – knowledge depth and knowledge breadth. Thus, future research could include details in their models to enhance understanding of such phenomenon. **Third**, our study collected data from U.S. state government IT departments. However, there are many other public sectors and geographical contexts. Future research could collect data from other public sectors such as non-profit organizations or other geographical contexts such as other countries to further test our model. **Finally**, we collected cross-sectional survey data to test our model. Even though we have included control variables to alleviate the concern on omitted variables and built the research model on a solid theory base to mitigate the concern on reverse causality, we cannot completely exclude the impact of endogeneity with the current research design, which prevents us from drawing causal inferences from our data. Future research could collect longitudinal data from multiple sources to validate the research model and address the potential endogeneity issues.

## Conclusion

The rise of big data era creates a highly dynamic business environment change. How to succeed in such fast-paced business environment is a critical issue to organizations. Since organization success is largely generated from organizational innovation, developing absorptive capacity is a one of the key factors that leads to organization success. Also, information systems can help managers access and analyze data from various sources, support business intelligence and analytics, and thereby provide insight into potential opportunities. Since information systems play a critical role in organization success. However, previous literature focused on the impacts of absorptive capacity in private sectors while ignoring examine the role of absorptive capacity in public sectors. In addition, few studies investigate the intertwined relationships among IS adoption, absorptive capacity, and organization performance. To address the research gap, we focus on the role of absorptive capacity in public sector and propose a research model to explore the relationship between IS adoption and organization performance from the absorptive capacity perspective. Using subjective data collected from 417 IS employees of 21 different state government in the United State, we reveal the intertwined relationships among IS adoption, organizational capabilities, IS-enabled absorptive capacity, and organization knowledge performance (manager level and employee level). These findings help us understand of how to leverage IS adoption to improve their organizational performance in public sector. By doing so, this study extended absorptive capacity in a brand-new context and provide actionable insights to state governments decision makers.

## Data availability statement

Datasets are available from the corresponding author upon reasonable request.

## Ethics statement

Ethical review and approval was not required for the study on human participants in accordance with the local legislation and institutional requirements. Written informed consent from the [patients/ participants OR patients/participants legal guardian/next of kin] was not required to participate in this study in accordance with the national legislation and the institutional requirements.

## Author contributions

All authors listed have made a substantial, direct, and intellectual contribution to the work and approved it for publication.

## Funding

This work is sponsored by Humanities and Social Science Fund of Ministry of Education of China (16YJA630025).

## Conflict of interest

The authors declare that the research was conducted in the absence of any commercial or financial relationships that could be construed as a potential conflict of interest.

## Publisher’s note

All claims expressed in this article are solely those of the authors and do not necessarily represent those of their affiliated organizations, or those of the publisher, the editors and the reviewers. Any product that may be evaluated in this article, or claim that may be made by its manufacturer, is not guaranteed or endorsed by the publisher.

## References

[ref1] Agramunt L. Berbel-Pineda J. Capobianco-Uriarte M. Casado-Belmonte M. (2020). Review on the relationship of absorptive capacity with Interorganizational networks and the internationalization process. Complexity. 2020, 1–20. doi: 10.1155/2020/7604579

[ref2] Ali M. Ali I. Al-Maimani K. A. Park K. (2018). The effect of organizational structure on absorptive capacity in single and dual learning modes. J. Innov. Knowl. 3, 108–114. doi: 10.1016/j.jik.2017.03.007

[ref3] Angst C. M. Agarwal R. (2009). Adoption of electronic health Records in the Presence of privacy concerns: the elaboration likelihood model and individual persuasion. MIS Q. 33, 339–370. doi: 10.2307/20650295

[ref4] Arena M. Cross R. Sims J. Uhl-Bien M. (2017). How to catalyze innovation in your organization. MIT Sloan Manag. Rev. 58, 38–48.

[ref5] Armstrong D. J. Liu Y. J. Riemenschneider C. K. (2015). How managers and workers see their world: Perceptions of the relationship between organizational capabilities and absorptive capacity in us state information system. ECIS 2015 Completed Research Papers.

[ref6] Barrett K. Greene R. (2008). Grading the states: the mandate to measure. Governing 21, 24–95.

[ref909] Boaden R. Lockett G. (1991). Information technology, information systems and information management: definition and development. Eur. J. Inf. Syst. 1, 23–32., PMID: 19121140

[ref7] Bolívar-Ramos M. T. García-Morales V. J. Martín-Rojas R. (2013). The effects of information technology on absorptive capacity and Organisational performance. Tech. Anal. Strat. Manag. 25, 905–922. doi: 10.1080/09537325.2013.823152

[ref8] Carte T. A. Russell C. J. (2003). In pursuit of moderation: nine common errors and their solutions. MIS Q. 27, 479–501. doi: 10.2307/30036541

[ref9] Chang S. Gong Y. P. Way S. A. Jia L. D. (2013). Flexibility-oriented Hrm systems, absorptive capacity, and market responsiveness and firm innovativeness. J. Manag. 39, 1924–1951. doi: 10.1177/0149206312466145

[ref10] Chen C. J. (2004). The effects of knowledge attribute. Alliance Character. Absorp. Capacity Knowl. Transf. Perform. Manag. 34, 311–321. doi: 10.1111/j.1467-9310.2004.00341.x

[ref11] Choi S. (2014). Developing relationship-specific memory and absorptive capacity in Interorganizational relationships. Inf. Technol. Manag. 15, 223–238. doi: 10.1007/s10799-014-0181-5

[ref12] Cohen J. Cohen P. West S. G. Aiken L. S. (2003). Applied Multiple Regression/Correlation Analysis for the Behavioral Sciences (3rd Ed.), Mahwah, NJ: Lawrence Erlbaum.

[ref13] Cohen W. M. Levinthal D. A. (1989). Innovation and learning: the two faces of R & D. Econ. J. 99, 569–596. doi: 10.2307/2233763

[ref14] Cohen W. M. Levinthal D. A. (1990). Absorptive capacity: a new perspective on learning and innovation. Adm. Sci. Q. 35, 128–152. doi: 10.2307/2393553

[ref15] Davis F. D. (1989). Perceived usefulness, perceived ease of use, and user acceptance of information technology. MIS Q. 13, 319–340. doi: 10.2307/249008

[ref16] Dong J. Q. Yang C.-H. (2015). Information technology and organizational learning in knowledge alliances and networks: evidence from U.S. pharmaceutical industry. Inf. Manag. 52, 111–122. doi: 10.1016/j.im.2014.10.010

[ref17] Engelman R. M. Fracasso E. M. Schmidt S. Zen A. C. (2017). Intellectual capital, absorptive capacity and product innovation. Manag. Decis. 55, 474–490. doi: 10.1108/MD-05-2016-0315

[ref18] Fishbein M. Ajzen I. (1975). Belief, Attitude, Intention and Behavior: An Introduction to Theory and Research, Addison-Wesley: Reading, MA.

[ref19] Flor M. L. Cooper S. Y. Oltra M. J. (2018). External knowledge search, absorptive capacity and radical innovation in high-technology firms. Eur. Manag. J. 36, 183–194. doi: 10.1016/j.emj.2017.08.003

[ref20] Francalanci C. Morabito V. (2008). Is integration and business performance: the mediation effect of organizational absorptive capacity in Smes. J. Inf. Technol. 23, 297–312. doi: 10.1057/jit.2008.18

[ref21] Frank A. G. Ribeiro J. L. D. Echeveste M. E. (2015). Factors influencing knowledge transfer between NPD teams: a taxonomic analysis based on a sociotechnical approach. R&D Manag., 45Placeholder Text 1–22. doi: 10.1111/radm.12046

[ref22] Gao S. Yeoh W. Wong S. F. Scheepers R. (2017). A literature analysis of the use of absorptive capacity construct in IS research. Int. J. Inf. Manag., 37Placeholder Text 36–42. doi: 10.1016/j.ijinfomgt.2016.11.001

[ref23] Hair J. F. Hult G. T. M. Ringle C. Sarstedt M. (2016). A primer on Partial Least Squares Structural Equation Modeling, Thousand Oaks, CA: Sage.

[ref24] Haneda S. Ito K. (2018). Organizational and human resource management and innovation: which management practices are linked to product and/or process innovation? Res. Policy 47, 194–208. doi: 10.1016/j.respol.2017.10.008

[ref25] Harman H. H. (1976). Modern Factor Analysis, Chicago, IL: University of Chicago Press.

[ref26] Harvey G. Skelcher C. Spencer E. Jas P. Walshe K. (2010). Absorptive capacity in a non-market environment: a knowledge-based approach to analyse the performance of sector organizations. Public Manag. Rev. 12, 77–97. doi: 10.1080/14719030902817923

[ref27] He J. Butler B. S. King W. R. (2007). Team cognition: development and evolution in software project teams. J. Manag. Inf. Syst. 24, 261–292. doi: 10.2753/MIS0742-1222240210

[ref28] Henseler J. Ringle C. M. Sarstedt M. (2015). A new criterion for assessing discriminant validity in variance-based structural equation modeling. J. Acad. Mark. Sci. 43, 115–135. doi: 10.1007/s11747-014-0403-8

[ref29] Hodgkinson I. R. Hughes P. Hughes M. (2012). Absorptive capacity and market orientation in public service provision. J. Strateg. Mark. 20, 211–229. doi: 10.1080/0965254X.2011.643915

[ref30] Inkpen A. C. Tsang E. W. (2005). Social Capital, Networks, and Knowledge Transfer. Manag. Rev. 30, 146–165. doi: 10.5465/amr.2005.15281445

[ref31] Iyengar K. Sweeney J. R. Montealegre R. (2015). Information technology use as a learning mechanism: the impact of IT use on knowledge transfer effectiveness absorptive capacity, and Franchisee performance. MIS Quart. 39, 615–642. doi: 10.25300/MISQ/2015/39.3.05

[ref32] Jansen J. J. Van Den Bosch F. A. Volberda H. W. (2005). Managing potential and realized absorptive capacity: how do organizational antecedents matter? Acad. Manag. J. 48, 999–1015. doi: 10.5465/amj.2005.19573106

[ref33] Jimenez-Castillo D. Sanchez-Perez M. (2013). Nurturing employee market knowledge absorptive capacity through unified internal communication and integrated information technology. Inf. Manag. 50, 76–86. doi: 10.1016/j.im.2013.01.001

[ref34] Kostopoulos K. Papalexandris A. Papachroni M. Ioannou G. (2011). Absorptive capacity, innovation, and financial performance. J. Bus. Res. 64, 1335–1343. doi: 10.1016/j.jbusres.2010.12.005

[ref35] Kotha R. George G. Srikanth K. (2013). Bridging the mutual knowledge gap: coordination and the commercialization of university science. Acad. Manag. J. 56, 498–524. doi: 10.5465/amj.2010.0948

[ref36] Kwok S. H. Gao S. (2005). Attitude towards knowledge sharing behavior. J. Comput. Inf. Syst. 46, 45–51. doi: 10.1080/08874417.2006.11645882

[ref37] Lane P. J. Koka B. R. Pathak S. (2006). The reification of absorptive capacity: a critical review and rejuvenation of the construct. Acad. Manag. Rev. 31, 833–863. doi: 10.5465/amr.2006.22527456

[ref38] Lane P. J. Lubatkin M. (1998). Relative absorptive capacity and Interorganizational learning. Strateg. Manag. J. 19, 461–477. doi: 10.1002/(SICI)1097-0266(199805)19:5<461::AID-SMJ953>3.0.CO;2-L

[ref39] Lichtenthaler U. Ernst H. (2012). Integrated knowledge exploitation: the complementarity of product development and technology licensing. Strateg. Manag. J. 33, 513–534. doi: 10.1002/smj.1951

[ref40] Lichtenthaler U. Lichtenthaler E. (2009). A capability based framework for open innovation: complementing absorptive capacity. J. Manag. Stud. 46, 1315–1338. doi: 10.1111/j.1467-6486.2009.00854.x

[ref41] Lichtenthaler U. Lichtenthaler E. (2010). Technology transfer across organizational boundaries: absorptive capacity and Desorptive capacity. Calif. Manag. Rev. 53, 154–170. doi: 10.1525/cmr.2010.53.1.154

[ref42] Lindell M. K. Whitney D. J. (2001). Accounting for common method variance in Cross-sectional research designs. J. Appl. Psychol. 86, 114–121. doi: 10.1037/0021-9010.86.1.114 11302223

[ref43] Liu Y. Armstrong D. J. Riemenschneider C. (2018). The relationship between information systems (is) assets, organizational capabilities, and IS-enabled absorptive capacity in us state information technology departments. Commun. Assoc. Inf. Syst. 42, 125–146. doi: 10.17705/1CAIS.04206

[ref44] Liu H. Ke W. Wei K. K. Hua Z. (2013). The impact of it capabilities on firm performance: the mediating roles of absorptive capacity and supply chain agility. Decis. Support. Syst. 54, 1452–1462. doi: 10.1016/j.dss.2012.12.016

[ref45] Liu Y. Tang X. Bush A. (2021). Intra-platform competition: the role of innovative and refinement evolution in app success. Inf. Manag. 58:103521. doi: 10.1016/j.im.2021.103521

[ref46] Lublin J. (2003). Deep, surface and strategic approaches to learning. Centre for Teaching and Learning Good Practice in teaching and learning. Available at: http://www2.Warwick.Ac.Uk/Services/Ldc/Development/Pga/Ġntrotandl/Resources/2a_Deep_Surfacestrategic_Approaches_To_Learning (Accessed November 27, 2018).

[ref47] Lund Vinding A. (2006). Absorptive capacity and innovative performance: a human capital approach. Econ. Innov. New Technol. 15, 507–517. doi: 10.1080/10438590500513057

[ref48] MacKenzie S. B. Podsakoff P. M. Podsakoff N. P. (2011). Construct measurement and validation procedures in Mis and behavioral research: integrating new and existing techniques. MIS Q. 35, 293–334. doi: 10.2307/23044045

[ref49] Mahnke V. Pedersen T. Venzin M. (2005). The impact of knowledge management on MNC subsidiary performance: the role of absorptive capacity. Manag. Int. Rev. 45, 101–119.

[ref50] Malhotra A. Gosain S. El Sawy O. A. (2005). Absorptive capacity configurations in supply chains: gearing for partner-enabled market knowledge creation. MIS Q. 29, 145–187. doi: 10.2307/25148671

[ref51] Manfreda A. Kovacic A. Stemberger M. I. Trkman P. (2014). Absorptive capacity as a precondition for business process improvement. J. Comput. Inf. Syst. 54, 35–43. doi: 10.1080/08874417.2014.11645684

[ref52] Mannucci P. V. Yong K. (2018). The differential impact of knowledge depth and knowledge breadth on creativity over individual careers. Acad. Manag. J. 61, 1741–1763. doi: 10.5465/amj.2016.0529

[ref53] Marabelli M. Newell S. (2014). Knowing, power and materiality: a critical review and reconceptualization of absorptive capacity. Int. J. Manag. Rev. 16, 479–499. doi: 10.1111/ijmr.12031

[ref54] Matusik S. F. Heeley M. B. (2005). Absorptive capacity in the software industry: identifying dimensions that affect knowledge and knowledge creation activities. J. Manag. 31, 549–572. doi: 10.1177/0149206304272293

[ref55] McHugh M. (1997). The stress factor: another item for the change management agenda? J. Organ. Chang. Manag. 10, 345–362. doi: 10.1108/09534819710175866

[ref56] Micheli P. Schoeman M. Baxter D. Goffin K. (2012). New business models for public-sector innovation: successful technological innovation for government. Res. Technol. Manag. 55, 51–57. doi: 10.5437/08956308X5505067

[ref57] Moore G. C. Benbasat I. (1991). Development of an instrument to measure the perceptions of adopting an information technology innovation. Inf. Syst. Res. 2, 192–222. doi: 10.1287/isre.2.3.192

[ref58] Morris M. G. Dillon A. (1997). How user perceptions influence software use. IEEE Softw. 14, 58–65. doi: 10.1109/52.595956

[ref59] Nouri B. A. Ghorbani R. Soltani M. (2017). The effect of knowledge management on organizational innovation with the mediating role of organizational learning. J. Appl. Econ. Bus. Res. 7, 194–211.

[ref60] Nutt P. C. (2006). Comparing public and private sector decision-making practices. J. Public Adm. Res. Theory 16, 289–318. doi: 10.1093/jopart/mui041

[ref61] Paarlberg L. E. Lavigna B. (2010). Transformational leadership and public service motivation: driving individual and organizational performance. Public Adm. Rev. 70, 710–718. doi: 10.1111/j.1540-6210.2010.02199.x

[ref62] Pandey S. K. Rainey H. G. (2006). Public Managers' perceptions of organizational goal ambiguity: analyzing alternative models. Int. Public Manag. J. 9, 85–112. doi: 10.1080/10967490600766953

[ref63] Patel P. C. Kohtamäki M. Parida V. Wincent J. (2015). Entrepreneurial orientation-as-experimentation and firm performance: the enabling role of absorptive capacity. Strateg. Manag. J. 36, 1739–1749. doi: 10.1002/smj.2310

[ref64] Pavlou P. A. El Sawy O. A. (2006). From IT leveraging competence to competitive advantage in turbulent environments: the case of new product development. Inf. Syst. Res. 17, 198–227. doi: 10.1287/isre.1060.0094

[ref65] Pérez-López S. Alegre J. (2012). Information technology competency, knowledge processes and firm performance. Ind. Manag. Data Syst. 112, 644–662. doi: 10.1108/02635571211225521

[ref66] Pian Q. Y. Jin H. Li H. (2019). Linking knowledge sharing to innovative behavior: the moderating role of collectivism. J. Knowl. Manag. 23, 1652–1672. doi: 10.1108/JKM-12-2018-0753

[ref67] Piccoli G. (2007). Information Systems for Managers: Texts and Cases New York: Wiley Publishing.

[ref68] Podsakoff P. M. MacKenzie S. B. Lee J.-Y. Podsakoff N. P. (2003). Common method biases in behavioral research: a critical review of the literature and recommended remedies. J. Appl. Psychol. 88, 879–903. doi: 10.1037/0021-9010.88.5.879 14516251

[ref69] Riemenschneider C. K. Allen M. W. Armstrong D. J. Reid M. F. (2010). Potential absorptive capacity of state IT departments: a comparison of perceptions of CIOs and IT managers. J. Organ. Comput. Electron. Commer. 20, 68–90. doi: 10.1080/10919390903482325

[ref70] Roberts N. Campbell D. E. Vijayasarathy L. R. (2016). Using information systems to sense opportunities for innovation: integrating Postadoptive use behaviors with the dynamic managerial capability perspective. J. Manag. Inf. Syst. 33, 45–69. doi: 10.1080/07421222.2016.1172452

[ref71] Roberts N. Galluch P. S. Dinger M. Grover V. (2012). Absorptive capacity and information systems research: review synthesis, and directions for future research. MIS Q. 36, 625–648. doi: 10.2307/41703470

[ref72] Rutz O. J. Watson G. F. (2019). Endogeneity and marketing strategy research: an overview. J. Acad. Mark. Sci. 47, 479–498. doi: 10.1007/s11747-019-00630-4

[ref73] Sarstedt M. Hair J. F. Cheah J. H. Becker J. M. Ringle C. M. (2019). How to specify, estimate, and validate higher-order constructs in PLS-SEM. Australas. Mark. J. 27, 197–211. doi: 10.1016/j.ausmj.2019.05.003

[ref74] Setia P. Patel P. C. (2013). How information systems help create Om capabilities: consequents and antecedents of operational absorptive capacity. J. Oper. Manag. 31, 409–431. doi: 10.1016/j.jom.2013.07.013

[ref75] Sivo S. A. Saunders C. Chang Q. Jiang J. J. (2006). How low should you go? Low response rates and the validity of inference in is questionnaire research. J. Assoc. Inf. Syst. 7, 351–414. doi: 10.17705/1jais.00093

[ref76] Sobel M. E. (1982). Asymptotic confidence intervals for indirect effects in structural equation models. Sociol. Methodol. 13, 290–312. doi: 10.2307/270723

[ref77] Somers T. M. Nelson K. G. (2004). A taxonomy of players and activities across the ERP project life cycle. Inf. Manag. 41, 257–278. doi: 10.1016/S0378-7206(03)00023-5

[ref78] Teigland R. Di Gangi P. M. Flaten B. T. Giovacchini E. Pastorino N. (2014). Balancing on a tightrope: managing the boundaries of a firm-sponsored OSS community and its impact on innovation and absorptive capacity. Inf. Organ. 24, 25–47. doi: 10.1016/j.infoandorg.2014.01.001

[ref79] Todorova G. Durisin B. (2007). Absorptive capacity: valuing a reconceptualization. Acad. Manag. Rev. 32, 774–786. doi: 10.5465/amr.2007.25275513

[ref80] Trantopoulos K. von Krogh G. Wallin M. W. Woerter M. (2017). External knowledge and information technology: implications for process innovation performance. MIS Q. 41, 287–300. doi: 10.25300/MISQ/2017/41.1.15

[ref81] Tsai W. P. (2001). Knowledge transfer in Intraorganizational networks: effects of network position and absorptive capacity on business unit innovation and performance. Acad. Manag. J. 44, 996–1004. doi: 10.2307/3069443

[ref82] Tu Q. Vonderembse M. A. Ragu-Nathan T. S. Sharkey T. W. (2006). Absorptive capacity: enhancing the assimilation of time-based manufacturing practices. J. Oper. Manag. 24, 692–710. doi: 10.1016/j.jom.2005.05.004

[ref83] Van den Bosch F. A. J. Volberda H. W. de Boer M. (1999). Coevolution of firm absorptive capacity and knowledge environment: organizational forms and combinative capabilities. Organ. Sci. 10, 551–568. doi: 10.1287/orsc.10.5.551

[ref84] Van der Post W. De Coning T. Smit E. (1997). An Instrument to Measure Organizational Culture. South Afr. J. Bus. Manag. 28, 147–168.

[ref85] Winter S. G. (2003). Understanding dynamic capabilities. Strateg. Manag. J. 24, 991–995. doi: 10.1002/smj.318

[ref86] Zahra S. A. George G. (2002). Absorptive capacity: a review, reconceptualization, and extension. Acad. Manag. Rev. 27, 185–203. doi: 10.5465/amr.2002.6587995

[ref87] Zhu Y. Q. Chiu H. Holguin-Veras E. J. I. (2018). It is more blessed to give than to receive: examining the impact of knowledge sharing on sharers and recipients. J. Knowl. Manag. 22, 76–91. doi: 10.1108/JKM-06-2016-0218

[ref88] Zou T. Ertug G. George G. (2018). The capacity to innovate: a meta-analysis of absorptive capacity. Innovations 20, 87–121. doi: 10.1080/14479338.2018.1428105

