# Supplementary material for: Information systems adoption and knowledge performance: An absorptive capacity perspective
Source: Front Psychol. 2023 Jan 10;13:1062780. doi: 10.3389/fpsyg.2022.1062780 (PMC9878606; doi:10.3389/fpsyg.2022.1062780)
Supplement: Supplementary file 1 [file Table_1.docx]

# Appendix

| **Construct** | **Items** | **Source** |
| --- | --- | --- |
| Employee Knowledge Performance | **WK1**: The general knowledge level of our first-line IT employees is high | (Tu et al., 2006) |
|  | **WK2**: The overall technical knowledge of our first-line IT employees is high |  |
|  | **WK3:** The general educational level of our first-line IT employees is high |  |
|  | **WK4:** The overall job competence of our first-line IT employees is high |  |
| Manager Knowledge Performance | **MK1:** The knowledge of our managers is adequate when making strategic management decisions | (Tu et al., 2006) |
|  | **MK2:** The knowledge of our managers is adequate when dealing with new technologies |  |
|  | **MK3:** The knowledge of our managers is adequate when managing daily operations |  |
|  | **MK4:** The knowledge of our managers is adequate when solving technical problems |  |
| Socialization Capabilities | **SC1:** The IT department’s mission is clear to almost everyone who works here | (Pandey and Rainey, 2006; Van der Post et al., 1997) |
|  | **SC2:** It is easy to explain the IT department’s goals to outsiders |  |
|  | **SC3**: The IT department has clearly defined goals |  |
|  | **SC4**: The IT department has strong values that are widely shared by its members. |  |
|  | **SC5**: IT employees have a clear understanding of what the department’s values and philosophies are. |  |
|  | **SC6**: There is little that binds members of the IT department to one another. |  |
|  | **SC7**: The IT department consistently makes employees aware of how they are expected to behave at work. * |  |
|  | **SC8**: Managers seldom communicate to employees what the IT department’s values and philosophies are |  |
|  | **SC9**: Managers seldom do anything that shows employees what is important for the IT department’s long-term success. |  |
| Coordination Capabilities | **COORD1:** We ensured that our work tasks (activities, designs, reports) fit together very well. | (Pavlou and El Sawy, 2006) |
|  | **COORD2:** Overall, our project team was well coordinated. |  |
|  | **COORD3:** We ensured that the output of our work was synchronized with the work of others. |  |
|  | **COORD4:** We ensured that the output of our work was of a form useful to others when needed (the right thing at the right time). |  |
|  | **COORD5:** We ensured an appropriate allocation of resources (e.g., information, time, reports) within our project team. |  |
|  | **COORD6:** Project team ensured a fair sharing of resources. |  |
|  | **COORD7:** Project team members were assigned to tasks commensurate with their task-relevant knowledge and skills. |  |
|  | **COORD8:** We ensured that there was compatibility between project team members expertise and work processes. |  |
| Web 2.0-enabled Absorptive Capacity | **AS1WEB2**: The IT department is able to recognize the value of knowledge received regarding Web 2.0. | (Kwok and Gao, 2005) |
|  | **AS2WEB2**: The IT department is able to assimilate knowledge received regarding Web 2.0 and turn it into its own knowledge base. |  |
|  | **AS3WEB2**: The IT department is able to apply the knowledge received regarding Web 2.0 to solve business problems. |  |
| SOA-enabled Absorptive Capacity | **AS1SOA**: The IT department is able to recognize the value of knowledge received regarding Service Oriented Architecture. | (Kwok and Gao, 2005) |
|  | **AS2SOA**: The IT department is able to assimilate knowledge received regarding Service Oriented Architecture and turn it into its own knowledge base. |  |
|  | **AS3SOA**: The IT department is able to apply the knowledge received regarding Service Oriented Architecture to solve business problems. |  |
| CRM-enabled Absorptive Capacity | **AS1CRM:** The IT department is able to recognize the value of knowledge received regarding Customer Relationship Management (CRM) technology. | (Kwok and Gao, 2005) |
|  | **AS2CRM**: The IT department is able to assimilate knowledge received regarding Customer Relationship Management (CRM) technology and turn it into its own knowledge base. |  |
|  | **AS3CRM**: The IT department is able to apply the knowledge received regarding Customer Relationship Management (CRM) technology to solve business problems. |  |
| SOA Adoption | Please identify where your IT department is in the process of adopting the technologies listed below using the following scale: Service Oriented Architecture | (Somers and Nelson, 2004) |
| CRM Adoption | Please identify where your IT department is in the process of adopting the technologies listed below using the following scale: Customer Relationship Management (CRM) technology | (Somers and Nelson, 2004) |
| Web 2.0 Adoption | Please identify where your IT department is in the process of adopting the technologies listed below using the following scale: Web 2.0 | (Somers and Nelson, 2004) |
| * Deleted item All items excluding SOA, CRM, Web 2.0 Adoption used the anchors (1 = strongly disagree, 7 = strongly agree).  SOA, CRM, Web 2.0 Adoption used the following anchors:  1 = Not considering adopting the technology 2 = Initiation (considering adoption of the technology) 3 = Adoption decision made 4 = Adaptation (initial implementation of the technology) 5 = Acceptance (employees are urged to commit to using the technology) 6 = Routinization (use of the technology is encouraged as a normal activity) 7 = Infusion (increased effectiveness obtaining by using the technology) | | |
